# Study on the Effect of Magnesium Chloride-Modified Straw Waste Biochar on Acidic Soil Properties

**DOI:** 10.3390/molecules29143268

**Published:** 2024-07-10

**Authors:** Zhigao Liu, Yuhang Dai, Tianyi Wen, Penglian Wei, Yunlin Fu, Mengji Qiao

**Affiliations:** 1 College of Resources, Environment and Materials, Guangxi University, Nanning 530004, China; lzgk18@gxu.edu.cn (Z.L.); 18014389048@163.com (Y.D.); 2 College of Forestry, Guangxi University, Nanning 530004, China; fylin@126.com (Y.F.); qiaomengji1982@163.com (M.Q.); 3 State Key Laboratory of Featured Metal Materials and Life-Cycle Safety for Composite Structures, Guangxi University, Nanning 530004, China

**Keywords:** plant straw, biochar, soil chemical properties, indoor soil simulation experiment, Mg-modified

## Abstract

Soil biochar is a kind of organic matter rich in carbon, which is of great significance in soil fertility improvement, fertilizer type innovation and greenhouse gas emission reduction. In this paper, Mg-modified biochar was prepared by thermal cracking using rice straw and corn straw as raw materials. The Mg-modified biochar and unmodified biochar were fully mixed with prepared soil samples at the addition amounts of 0.5% (*w*/*w*), 1% (*w*/*w*) and 2% (*w*/*w*), respectively, and then simulated indoor soil cultivation experiments were carried out. The effects of magnesium ion-modified biochar and non-modified biochar on soil chemical properties and the effects of different amounts of biochar on soil properties were studied. The results showed that the yield of Mg-modified biochar from rice straw and corn straw, prepared by pyrolysis, was 65%, and the ash content was large. The pH of MG-modified corn stalk biochar (MCBC) is weakly basic (8.55), while the pH of MG-modified rice stalk biochar (MRBC) is basic (10.1), and their internal structures are slightly different. After the application of biochar prepared from rice straw and maize stover, soil indicators were determined. Compared to the control, the chemical properties of the treated soil samples were significantly improved, with an increase in soil pH, an increase in the content of effective nutrients, such as fast-acting potassium, fast-acting phosphorus and alkaline dissolved nitrogen, and an increase in the content of the total phosphorus and total nitrogen, as well as an increase in the content of organic matter. The Mg-modified biochar was generally superior to the unmodified biochar in improving soil fertility, at the same addition level. It was also found that the rice-straw biochar performed better than the corn-stover biochar and had a more obvious effect on soil improvement in terms of fast-acting potassium, ammonium nitrogen, nitrate nitrogen, total phosphorus and total nitrogen contents.

## 1. Introduction

Climate change is one of the major challenges at present, and the carbon cycle plays an important role in mitigating climate change [1]. At present, the method of increasing the carbon cycle by decarbonizing soil to mitigate climate change has been widely discussed [2,3]. At the same time, China is a big agricultural country, and crop-straw resources are rich. However, with the efficient cultivation of farmland and the continuous improvement in transportation costs, most of the straw is directly burned [4]. Therefore, if the straw is properly treated and used to modify the soil, it can bring economic benefits, while reducing environmental pollution and improving climate change. Recently, biomass raw materials (wood, bamboo, straw, etc.) have been used to research and develop multi-functional carbon materials [5,6]. They are widely used in supercapacitors [7], soil amendments, adsorption [8] and water treatment [9].

As one of the important soil amendments, biochar has been paid more and more attention to. Therefore, an increasing number of studies are using physical or chemical methods to modify or activate the structure of biochar in order to meet its application requirements or enhance its performance in specific fields [10]. It can be synthesized from a variety of carbon-based raw materials, including woody biomass, crop straw, animal manure and urban polluted mud, through pyrolysis, which reduces environmental pollution and realizes the comprehensive utilization of resources [11,12,13]. In recent years, a substantial body of research has consistently demonstrated that waste biomass can be transformed into biochar by pyrolysis, which makes use of the stable carbon composition of biochar itself and uses it to isolate CO_2_, N_2_O and CH_4_, thereby reducing the greenhouse effect and promoting soil carbon solidification [14,15,16], achieving a sustainable climate-smart agriculture [17]. In addition, biochar also has the advantages of rich surface functional groups, a large specific surface area, and strong cation exchange ability [18]. Metal is often used to modify biochar. This method can attach corresponding metal cations to the surface of biochar, which can enhance the physical and chemical reactions with nitrogen and phosphorus, thus enhancing the adsorption performance of nitrogen and phosphorus [19].

Mg is often used for biochar modification because of its abundant reserves and non-toxic and strong affinity for anions [20]. Wu et al. [10] modified peanut-shell biochar with magnesium oxide and found that the application of magnesium-modified biochar could increase the available phosphorus content of saline–alkali soil and reduce soil phosphorus leaching, indicating that magnesium-oxide biochar as a soil amendment could not only improve crop productivity, but also increase rice crop yield. Shan et al. [21] applied Mg-modified peanut-hull biochar to soil for the remediation of cadmium-contaminated soil, and the results showed that magnesium-modified biochar effectively increased the soil pH, reduced soil cadmium bioefficacy and cadmium content in plant vegetation. Studies have shown that the chemical activity of biochar can be enhanced by adding appropriate magnesium elements [22], thereby increasing the available phosphorus concentration of soil, increasing the photosynthesis and nutrient absorption of plants, improving the water holding capacity of soil and further increasing the yield of crops [10,23]. The application of modified biochar to soil can also provide nutrients for microorganisms, increasing the microbial reservoir of carbon and nitrogen in the soil, which is of great significance for improving soil–nutrient dynamics and maintaining soil organic carbon pools [24,25]. Biochar in soil remediation can remove metal ions and persistent organic pollutants in addition to soil improvement. Biochar’s own porous structure, hydroxyl and carbonyl properties would contribute to the degradation of heavy metal pollutants. Biochar, as a high-quality and low-cost amendment, can be used as an alternative material for ameliorating heavy metal-contaminated soils [26].

In general, the properties of biochar exhibit variations based on the types of raw materials and preparation methods, so the incorporation of biochar into the soil will elicit diverse impacts on soil chemistry and plant growth [27,28,29]. The soil in Guangxi is mainly acidic, and the most prominent shortcoming is the low concentration of available magnesium in the soil. The compound, which constitutes a vital element of plant chlorophyll and assumes a pivotal role in the process of plant growth, holds significant importance [17]. The growth of crops has certain requirements for the concentration of magnesium in soil, so it is very important to explore green and efficient soil amendments. The Mg-modified biochar exhibits a significantly enhanced specific surface area and abundant pore structure. However, limited research has been conducted on the impact of Mg-modified biochar on soil chemical properties. Therefore, this study prepared Mg-modified rice-straw and corn-straw biochar by modifying rice-straw and corn-straw biochar, intending to explore the effects of Mg-modified biochar on soil chemical properties. It applied rice-straw and corn-straw biochar and their modified biochar to soil in different proportions to explore the effects of different amounts of biochar on improved soil chemical properties. Through the culture experiment in a constant temperature incubator for 150 days, the similarities and differences of each index under different treatments were compared, the effects of Mg-modified rice-straw and corn-straw biochar on soil chemical and biological properties were discussed, and the properties, effects and optimal utilization ratio of magnesium ion-modified biochar were studied. This experiment will help to further explore the potential significance and value of biochar, promote the production of biochar for specific purposes and provide a certain theoretical basis for the production upgrade of forestry and agricultural products.

## 2. Results

### 2.1. Modified Biochar Yield, pH, Ash Content

The basic indexes of Mg-modified biochar for rice straw and corn straw are shown in Table 1. As can be seen from Table 1, the yield and ash content of MRBC are higher than those of MCBC (with MRBC having a 12.2% higher yield and 229.4% higher ash content), and the pH of both is alkaline, with MRBC being more alkaline. It can be seen that the rice-straw and corn-straw biochar samples modified by magnesium ions are mainly alkaline or weakly alkaline.

### 2.2. Effect on Soil pH

**Figure 1:**
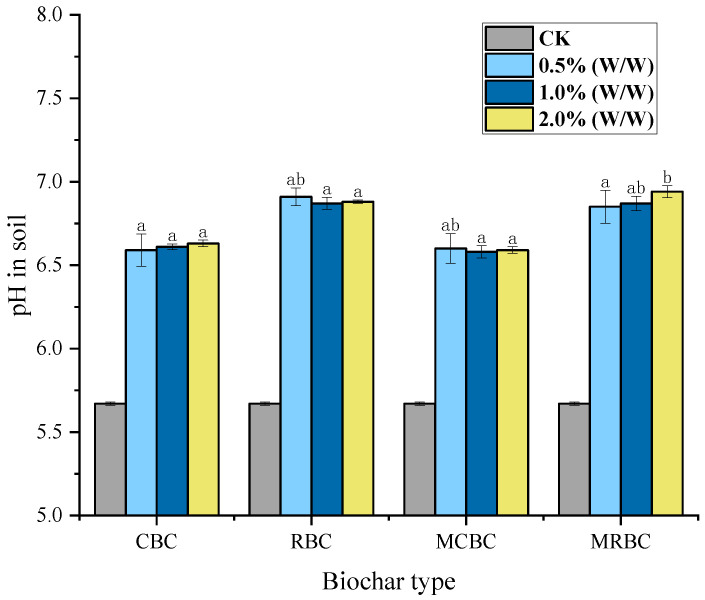
The pH of soil samples under different treatments. Note: The bars in the graphs show the mean ± standard deviation of the indicators; lowercase letters indicate the differences in the effects of different Mg-modified biochar on soil chemical properties (*p* < 0.05), as below.

The pH of group CK measured by a potentiometer is 5.67, and the soil pH of each biochar addition treatment is shown in Figure 1. In this experiment, the application of biochar to soil samples can improve soil acidity. RBC and MRBC (rice-straw biochar) significantly increased the soil pH value compared to CBC and MCBC (corn-straw biochar) at each addition level, increasing the soil pH value from 5.67 to 6.87–6.94. Compared to corn-stalk biochar, the increase from 5.67 to 6.58–6.63 was 0.24–0.36 units.

### 2.3. Effect on Soil Available Potassium Content

**Figure 2:**
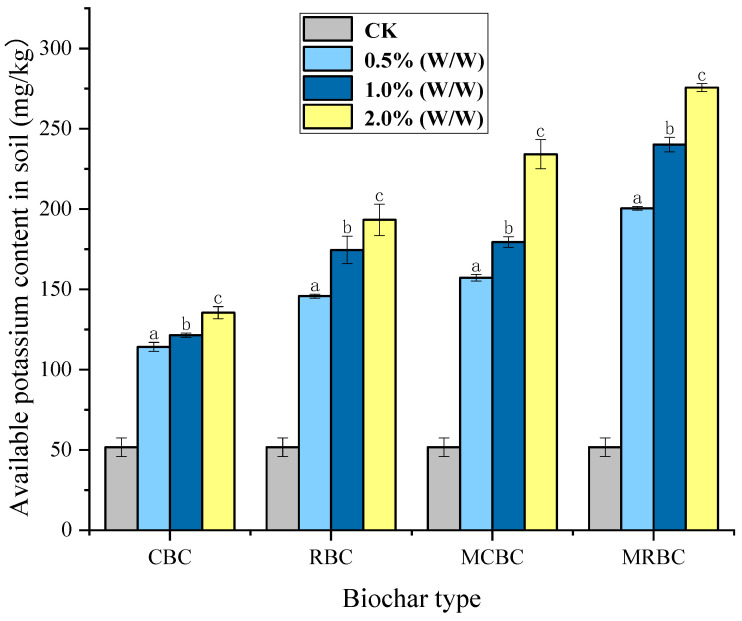
Available potassium content in soil samples under different treatments. Note: The bars in the graphs show the mean ± standard deviation of the indicators; lowercase letters indicate the differences in the effects of different Mg-modified biochar on soil chemical properties (*p* < 0.05), as below.

The obtained K^+^ formula is y = 0.0112x − 3.8092, R^2^ = 0.9998. After calculation using the formula, the content of soil available potassium determined is shown in Figure 2. As can be seen from the figure, compared to the control group, CK, the soil available potassium content of all treated samples with added biochar increased by more than 200%, and the available potassium content of the CK group was 51.73 mg/kg. The highest available potassium content in soil samples treated with rice-straw Mg-modified biochar MRBC was 275.62 mg/kg. Through a horizontal comparison, it was found that the effect of four kinds of biochar on increasing the content of available potassium was significantly different under the same addition amount. At each addition level, the effect of increasing the soil available potassium content was in the order of MRBC, MCBC, RBC and CBC from highest to lowest. Through longitudinal comparison, it was found that the content of available potassium increased with the increase in the addition amount of each kind of biochar.

### 2.4. Effect on Soil Available Phosphorus Content

**Figure 3:**
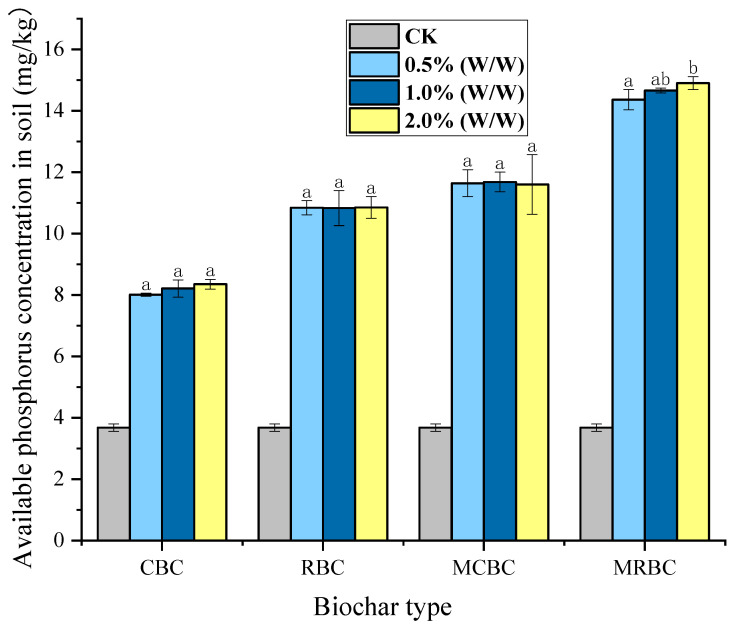
Available phosphorus content in soil samples under different treatments. Note: The bars in the graphs show the mean ± standard deviation of the indicators; lowercase letters indicate the differences in the effects of different Mg-modified biochar on soil chemical properties (*p* < 0.05), as below.

After processing the data, the standard curve y = 0.1341x + 0.0402, R^2^ = 0.9953 of the concentration and the absorbance of the standard phosphorus solution were obtained, and the available phosphorus content of each treatment group was calculated. As can be seen from Figure 3, the soil quick phosphorus content of the soil samples treated with added biochar increased by 122.6–297.8% compared to the control group, with the soil quick phosphorus of the CBC-treated samples being higher than that of the control group, CK, by 122.6%. The soil quick phosphorus of MCBC was higher than that of CK by 194.5%, and the soil quick phosphorus of RBC was higher than that of CK by 216.3%. MRBC showed the highest increase, at 297.8% higher than CK, i.e., the rice-straw biochar was more effective than the corn-stover biochar in increasing soil quick-acting phosphorus content, whether magnesium-modified or unmodified. The comparison from the point of view of different additions revealed that there was no significant difference, correlation or regularity between the different additions of either CBC, MCBC, RBC or MRBC.

### 2.5. Effect on Soil Alkali-Hydrolyzed Nitrogen Content

**Figure 4:**
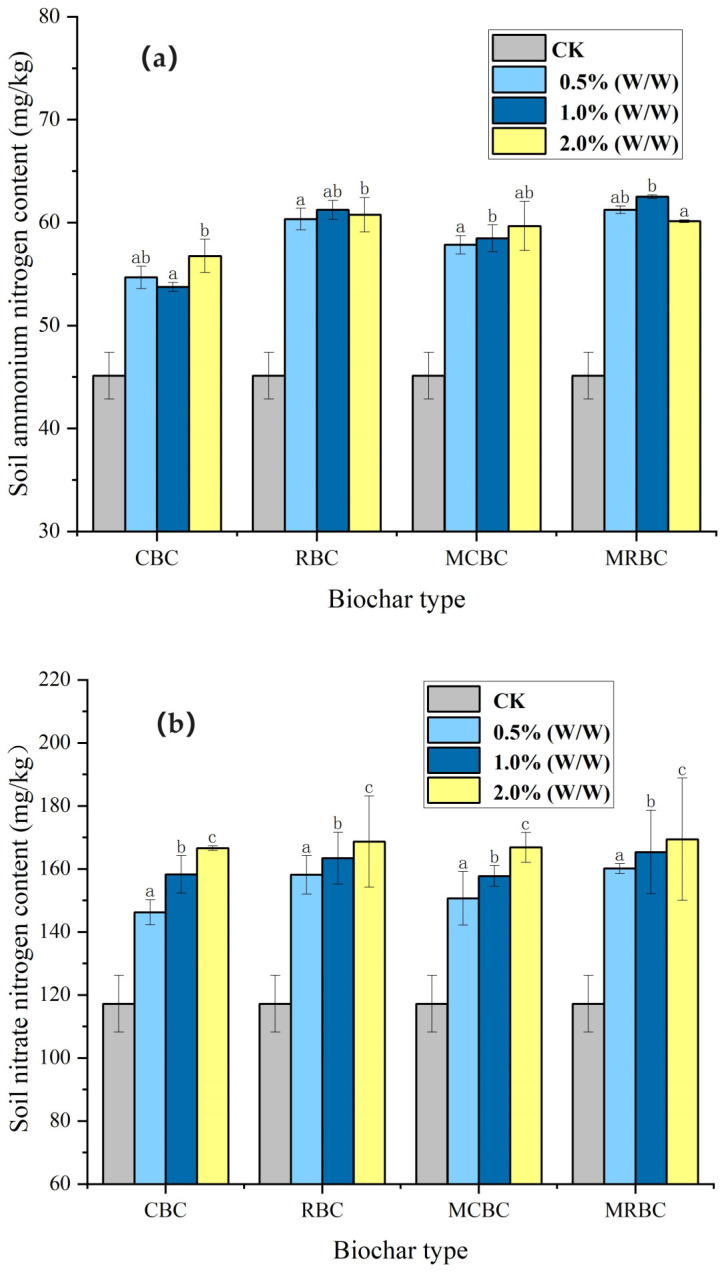
Content of ammonium nitrogen (**a**) and nitrate nitrogen (**b**) in soil samples under different treatments. Note: The bars in the graphs show the mean ± standard deviation of the indicators; lowercase letters indicate the differences in the effects of different Mg-modified biochar on soil chemical properties (*p* < 0.05), as below.

As can be seen from Figure 4, the ammonium nitrogen content was 45.13 mg/kg, and the nitrate-nitrogen content was 117.2 mg/kg in the CK group. In this study, the ammonium nitrogen content of the soil samples supplemented with soil biochar was increased by 22%, 29.98%, 34.67%, and 35.81% compared to that of the CBC, RCBC, MCBC and MRBC in the CK group, respectively, and the soil samples supplemented with soil biochar nitrate-nitrogen content increased by 33.98%, 35.16%, 39.43% and 40.75% compared to the CBC, RBC, MCBC and MRBC of the CK group, respectively. From the results, the overall improvement effect of rice-straw biochar was better than that of corn-straw biochar.

It is evident from the chart analysis that the addition of biochar to soil resulted in a noticeable rise in both ammonium nitrogen and nitrate-nitrogen concentrations. The difference between the treatments and the CK group was significant (*p* < 0.05). In terms of the change in the content of soil ammonium nitrogen, there was no correlation or regularity between the amount of biochar added and the content of ammonium nitrogen. There was even a situation in which the ammonium nitrogen content of soil samples treated with CBC with 1% (*w*/*w*) addition was lower than that of the soil samples treated with 0.5% (*w*/*w*) and 2% (*w*/*w*) CBC. However, the ammonium nitrogen content of the soil samples treated with RBC with 1% (*w*/*w*) addition was higher than that of the 0.5% (*w*/*w*) and 2% (*w*/*w*) treatments. This situation also appears in the MCBC, MRBC and other treatments, so there is no linear relationship between the amount of biochar added and the content of ammonium nitrogen. In contrast, in each biochar treatment, the nitrate-nitrogen content increased with the increase in biochar addition, and the increase of 1% (*w*/*w*) was the largest.

### 2.6. Effect on Total Nitrogen and Total Phosphorus Contents in Soil

#### 2.6.1. Soil Total Phosphorus

**Figure 5:**
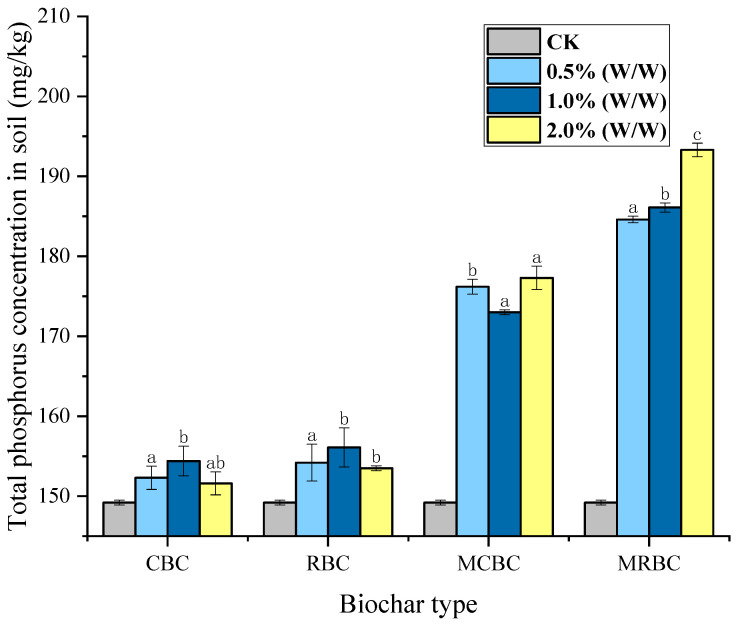
Total phosphorus nutrient content of biochar treated with different addition amounts. Note: The bars in the graphs show the mean ± standard deviation of the indicators; lowercase letters indicate the differences in the effects of different Mg-modified biochar on soil chemical properties (*p* < 0.05), as below.

The overall phosphorus concentration of the CK group was 149.2 mg/kg. Other test results are shown in Figure 5. The total phosphorus content in the soil treated with unmodified biochar CBC and RB basically showed no significant increase, and the difference compared to the control CK was only 2.5% and 4%. The total phosphorus content in the soil samples treated with magnesium-modified biochar MCBC and MRBC increased by 17.4% and 26.2%, respectively. However, a pattern between the amount of addition and the increase in total phosphorus could not be obtained. Even in the MCBC treatment, the total phosphorus content of the samples with 1% (*w*/*w*) addition was lower than that of the treatment with 0.5% (*w*/*w*) addition, and, therefore, it did not reach a statistically significant level.

#### 2.6.2. Soil Total Nitrogen

**Figure 6:**
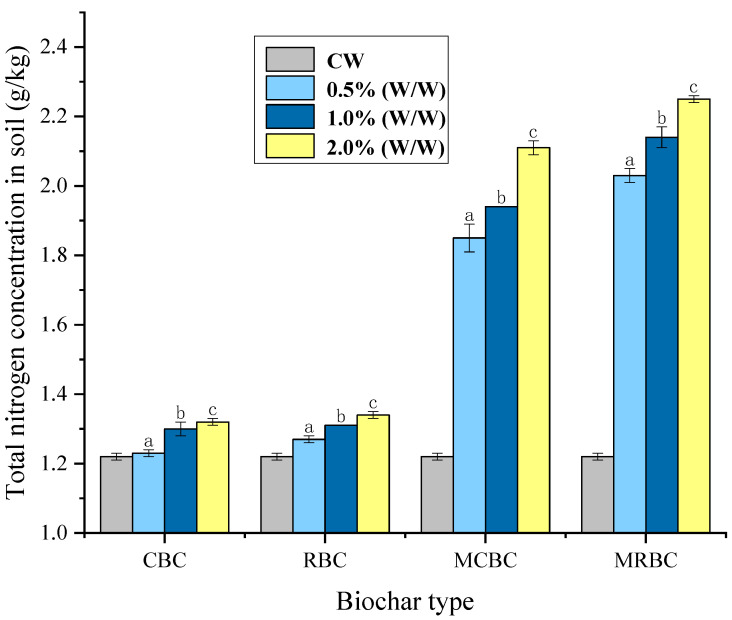
Total nitrogen nutrient content across different addition amounts of biochar treatment. Note: The bars in the graphs show the mean ± standard deviation of the indicators; lowercase letters indicate the differences in the effects of different Mg-modified biochar on soil chemical properties (*p* < 0.05), as below.

The overall nitrogen concentration within the CK group was 1.22 (g/kg), and other measurement results are shown in Figure 6. From the figure, it can be seen that although the total nitrogen content of soil samples was increased in all treatments, with the CBC, MCBC, RBC and MRBC increasing by 5.1%, 61.2%, 7.1% and 75.4%, respectively, the effect of unmodified rice-straw biochar and that of unmodified maize-stover biochar on the increase in the total nitrogen content was very small, whereas the effect of magnesium-modified biochar on the increase in the total nitrogen content was more significant. Charcoal modified with magnesium exhibited a more significant improvement effect.

Through longitudinal comparison, it was found that with different additions of Mg-modified biochar, the content of total nitrogen increased continuously with the increase in biochar additions, with the MCBC increasing the improvement value from 54.1% to 74.8. The MRBC increased the improvement value from 69.1% to 87.6%. It is evident that the performance of the MRBC surpasses that of the MCBC in terms of its impact on Mg-modified biochar. The improvement effect is better, but when the linear relationship was simulated by regression, R^2^ = 0.7632 was obtained, and the linear correlation between the added amount and the value of total nitrogen improvement was not significant.

### 2.7. Effect on Soil Organic Matter Content

**Figure 7:**
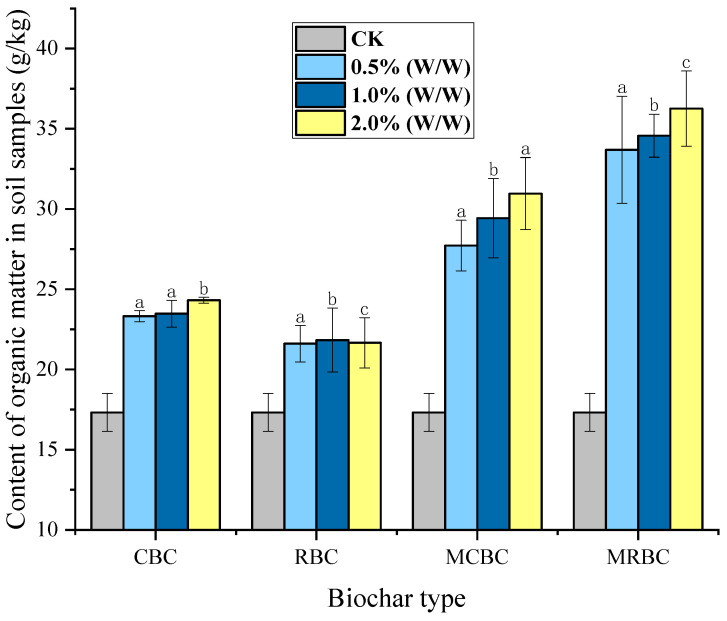
Content of organic matter in soil samples under different treatments. Note: The bars in the graphs show the mean ± standard deviation of the indicators; lowercase letters indicate the differences in the effects of different Mg-modified biochar on soil chemical properties (*p* < 0.05), as below.

In the determination using the hydrated thermal potassium dichromate oxidation-colorimetric method, the standard curve for the concentration of potassium permanganate and absorbance was obtained as y = 0.0117x + 0.0415, R^2^ = 0.9963, indicating a good correlation, and the composition of organic matter in the CK group was calculated to be 17.32 g/kg, which was used to obtain the content of organic matter in each treatment group, as shown Figure 7. Compared to the control group, the organic matter content in the treatment group supplemented with biochar was significantly higher, in which the CBC, MCBC, RBC and MRBC were 36.8%, 67.6%, 25.3% and 101.2% higher than the CK in the control group, respectively. In addition, there were no statistically significant differences observed in the treatment outcomes of biochar at concentrations of 0.5%, 1% and 2% (*w*/*w*). However, the organic matter content showed a small increase from 0.5% (*w*/*w*) to 2% (*w*/*w*) with the increase in soil biochar addition. The 1% addition of CBC resulted in a 0.6% higher organic matter content compared to the 0.5% (*w*/*w*) addition of CBC, and the 2% addition of CBC resulted in a 3.5% higher organic matter content compared to the 1% (*w*/*w*) addition. For the same comparison, the enhancement values were 6.1% and 5.2% for different additions of MCBC, 0.5% and −1.0% for different additions of RBC and 2.6% and 4.9% for different additions of MRBC. The effect of modified biochar (MCBC, MRBC) on the improvement of soil organic matter compared to unmodified biochar (CBC, RBC) was more pronounced with the increase in the amount of addition. The incorporation of biochar significantly augmented the soil’s organic matter content, showing a small increase from 0.5% (*w*/*w*) to 2% (*w*/*w*) with the increase in soil biochar addition and, in general, the improvement of the MRBC was better than that of the MCBC.

## 3. Discussion

The improvement effect of biochar on soil chemical properties is closely related to the structure and physicochemical properties of the biochar itself, which are mainly determined by the selection of raw materials, preparation methods and preparation conditions. The selection of raw materials significantly influences the characteristics of biochar produced, leading to notable variations in the properties observed among different feedstocks [30,31].

Clarifying the different characteristics of the effects of Mg-modified biochar from rice straw and corn straw on soil and exploring the variation rules of the effects of different addition amounts can provide references for preparing and screening biochar in the process of forestry production, assisting forest workers in screening raw materials and evaluating appropriate addition amounts in a targeted manner. In this study, two kinds of biochar from different raw materials (rice straw and corn straw) were prepared, and the variation rules of the biochar’s yield, pH and ash content, as well as the effects of its addition amount on soil chemical properties were investigated.

In China’s agricultural production, soil acidification has become a more prominent problem due to rapid development. Many agriculturalists are also looking for sustainable ways to improve acidified soil, and improving soil acidification is also a top priority in ecological conservation.

Through the significance test, it was found that there was no significant difference in the soil’s pH content between different types of biochar supplementation (*p* > 0.05), and there was no good correlation regularity. Wu Min et al. [32] studied the improvement effects of coconut charcoal, cane charcoal and other biochar treatments on soil acidity. After applying these treatments to soil, they finally found the following: the pH value of all soil-treated samples increased significantly when the biochar addition rate was in the range of 1.5~7.5% (*w*/*w*). With the increase in biochar addition rate, the soil pH value of coconut carbon increased from 4.8 to 5.5~7.2, and the soil pH value of cane carbon increased from 5.0~5.7. In addition, it was found that the soil pH increased more in the samples treated with high biochar addition than in the samples treated with low biochar addition. Mg-modified biochar contains many alkaline substances, such as carbonate (CaCO_3_), carboxyl (COO-) and phosphoric (PO_4_^3−^) during pyrolysis. These alkaline substances used in acidic soil can neutralize soil acidity and increase soil pH [33,34]. Mg-modified biochar can increase the soil pH value and contains Ca^2+^, K^+^ and Mg^2+^, which are related to the exchange of H^+^ and Al^3+^ in the soil [35]. However, the ability of biochar to improve the soil pH value in this study was not correlated with the different amounts of biochar added, the final sintering temperature or the holding time, which may be due to the insufficient gradient between the different addition amounts in this experiment and the use of a 0.5 mol/L magnesium chloride solution impregnation, which is unable to cause regular changes in pH.

Potassium is one of the three essential nutrients for plant growth and development, and it has an important impact on the necessary physiological activities and normal growth and development of plants [36]. In this study, the situation in which the available potassium content also increased with the increase in the added amount of biochar was consistent with the increase in the added amount of biochar. The results of the significance test (*p* < 0.001) also showed that the available potassium content of the soil was significantly increased by biochar, within the range of 0.5% (*w*/*w*)~2% (*w*/*w*); with the increase in the added amount of biochar, the content of available potassium in soil was significantly increased. The amount of available potassium also increased. When Mg-modified biochar is applied to soil, it can directly supplement soil potassium through the release of potassium ions contained in it and improve the availability of potassium in soil through the reduction in soil bulk density, enhancement of soil porosity, optimization of soil water retention capacity, adjustment of soil pH level and increase in cation exchange capacity [37].

Phosphorus is an important nutrient element in forest ecosystems, which is involved in various growth and metabolism activities of plants, with available phosphorus being the most easily absorbed nutrient by plants in soil. Zhang et al. [38] found two important reasons why biochar increases the soil available phosphorus content: Biochar itself contains many minerals; that is, biochar itself contains many phosphorus elements, and phosphorus elements are easily released after being applied to the soil. The longer the action time of biochar in the soil, the more phosphorus elements in it are constantly released to supplement the soil phosphorus content. The structure of biochar itself, including its porosity and specific surface area, as well as other good physical properties, allows the phosphorus in the soil to be adsorbed by the pores of the biochar, which helps to better maintain the soil’s effective phosphorus content. Based on the above two reasons, the quick-acting phosphorus content in the soil increases. In this study, it was found that from the perspective of different additions, when comparing different additions of CBC, MCBC, RBC, MRBC, there was no significant difference between their additions, and there was no significant correlation or regularity (*p* > 0.05). In other experiments, it was also found that the soil quick-acting phosphorus content was lower after treatment with high additions of biochar compared to low additions.

The nitrogen source in crops mainly comes from the absorption of nitrate and ammonium nitrogen in the soil, so the determination of the content of nitrate and ammonium nitrogen in the soil can be used as one of the important indicators for improving soil fertility by biochar. Soil alkali-hydrolyzed nitrogen or hydrolyzed nitrogen is composed of both inorganic nitrogen (ammonium nitrogen and nitrate nitrogen) and organic nitrogen that can be easily broken down, such as amino acids, ammonium acyl and proteins that are easily hydrolysable. When soil is treated with lye, organic nitrogen and ammonium nitrogen, which are readily hydrolyzed, undergo conversion to ammonia. Additionally, nitrate nitrogen is initially transformed into ammonium through the action of ferrous sulfate proteins. The ammonia was absorbed using boric acid and subsequently titrated with a standard acid in order to determine the concentration of hydrolyzed nitrogen. The results of this study showed that the application of biochar significantly improved the accumulation of ammonium nitrogen and nitrate nitrogen in the soil and increased the nitrogen supply capacity of the soil. This phenomenon may be attributed to the presence of a certain nitrogen content in biochar, or it may be because the solidification of microorganisms enhances the retention of nitrogen in the soil. The results of this study showed that the application of biochar significantly improved the accumulation of ammonium nitrogen and nitrate nitrogen in the soil and increased the nitrogen supply capacity of the soil, which was similar to the results of Gupta et al. [39] However, no correlation was found between the amount of soil biochar added and the improvement effects.

In this study, it was found that the content of total nitrogen increased with the increase in the additional amount of Mg-modified biochar. The main reason for the increase in the soil nitrogen content by biochar is that the strong adsorption capacity of biochar plays a role. The utilization of biochar in soil significantly improves the adsorption and retention capacity of soil nitrogen [39]. It can be seen that biochar can effectively reduce the loss of nitrogen in the soil, and the total amount of total nitrogen increases gradually with the increase in biochar addition. In addition, the internal structure of the magnesium-modified biochar is more distinctive, with the presence of a large number of voids, and both the specific surface area of the BET and the micropores are all increased, so the modified biochar is more effective in improving the total nitrogen content. Although the soils treated with unmodified biochar CBC and RBC did not exhibit a statistically significant increase in the total phosphorus content, there was a significant increase in the content of total phosphorus in the soil samples treated with magnesium-modified biochar MCBC and MRBC, but because the gradient of the additions did not reach a statistically significant correlation in terms of the change in the content of total phosphorus, it was not possible to obtain a pattern between the amount of additions and the increase in the total phosphorus.

Biochar plays a positive role in restoring soil fertility and improving soil productivity, and its possible mechanism can be explained through the following three aspects: First, biochar improves soil-ion exchange capacity, thus promoting the absorption of nutrients by plants. Secondly, biochar has a certain binding and retention effect on soil nutrients. Third, biochar can create a favorable environment conducive to the proliferation and activity of soil microorganisms [40], thus promoting the nutrient circulation of the soil ecosystem and playing an important role in maintaining soil quality and health [41]. The rice-straw and corn-straw Mg-modified biochar treatments were prepared by pyrolysis, and the yield was 65%. Its pH is alkaline (MRBC), and the ash content of biochar is large. The study by Qin et al. showed that rice-straw and maize-straw Mg-modified biochar has a good specific surface area, total pore volume, micropore volume and mesopore volume, and it is because of these conditions that biochar can improve soil properties [35].

It was found through experimental measurements that the pH, quick-acting potassium, quick-acting phosphorus, ammonium nitrogen, nitrate nitrogen, total phosphorus, total nitrogen and organic matter contents of the treated soils increased to a certain extent after the soil was treated with biochar, compared to the control group, with a small increase in the content of total phosphorus and high increases in all other indicators. This could be attributed to the alkaline properties of biochar, its high capacity for cation exchange and extensive surface area and porosity, along with the existence of functional groups, such as carboxyl groups, on its surface. These factors play a crucial role in determining the efficacy of biochar in enhancing soil fertility [42].

The major elemental compositions of MCBC, CBC, MRBC and RBC are C, H, N and O. The elemental carbon content of the modified biochar showed an increasing trend with the carbonization dimension. On the contrary, with increasing pyrolysis temperature, the H, N and O content of modified biochar decreased, which was mainly due to the loss of volatile components and dehydration of organic matter, as well as the breakage of weak bonds within the feedstock structure [35,43,44].

Among the soil indexes treated with biochar, only the total nitrogen content and organic matter content of the soil treated with Mg-modified biochar (MCBC and MRBC) increased with the increase in biochar addition. The contents of nitrate nitrogen and available potassium in the soil samples treated with unmodified biochar (CBC, RBC) and Mg-modified biochar (MCBC and MRBC) also increased with the increase in biochar addition. However, there is no significant correlation between the increase in pH, available phosphorus, ammonium nitrogen and total phosphorus content and the different addition amounts of biochar, which may be due to the narrow range of additions set in this experiment, with only three gradients, which requires further exploration. At the same time, through the comparison of biochar types and the analysis of the improvement effect on soil fertility, it was found that the overall improvement effect of Mg-modified biochar was better than that of unmodified biochar under the same additional amount. It was also found that the rice-straw biochar had better performance compared to the corn-straw biochar, and the rice-straw Mg-modified biochar (MRBC) was more effective than the corn-straw Mg-modified biochar in increasing the contents of available potassium, ammonium nitrogen, nitrate nitrogen, total phosphorus and total nitrogen. However, the effect of rice-straw unmodified biochar (RBC) was better than that of corn-straw Mg-modified biochar (MCBC) in increasing the content of available phosphorus, which may be related to the soil culture stage or soil pH change. Additional comparisons were made with the results of other experimental studies, and the comparisons are shown in Table S2. It was found that most of the indicators in the MRBC-modified soil were better than those in the MRBC-modified soil. However, the findings were opposite in terms of the total phosphorus content, probably due to the large difference in soil pH between the two soils.

## 4. Materials and Methods

### 4.1. Preparation of Modified Biochar

The agricultural wastes used in this experiment were corn straw and rice straw, with the rice straw collected from the experimental base farmland of the College of Agriculture, Guangxi University, while the corn straw was obtained from the farmland in Mashan County, Guangxi. The samples were taken on 6 November 2022, and 15 November 2022, respectively. After the sampling was completed, the rice and corn straw were dried in natural air and then crushed with a Multi-function grinding machine to obtain the powdered substance, which was dried and then passed through 80 mesh sieves. The sieved powder was kept in the prepared self-sealing bag and placed in the drier for use.

Similar to the experimental steps of Wang, J, the biochar used in this experiment was prepared through the oxygen-limited slow-cracking method [45], which was operated as follows: the dried straw powder was loaded into a quartz boat and subsequently positioned within the heating zone of the MFLGKDF406-12 tube furnace, and an amount of nitrogen was introduced into the furnace to ensure that the oxygen in the furnace was exhausted. The biochar was produced by subjecting it to pyrolysis at high temperatures in an oxygen-free environment. It was heated to 300 °C at a heating rate of 8 °C/min, and then the constant temperature was heated for 1 h, during which the nitrogen was always connected. The biochar obtained at this time was called precursor biochar. The precursor biochar prepared from corn straw was named CBC-300, and the precursor biochar prepared from rice straw was named RBC-300. Following the procedure in the LI, S’s experiment, the precursor biochar was added into magnesium chloride solution with a concentration of 0.5 mol/L [46], in which each 10 g of precursor biochar was mixed with 500 mL of a magnesium chloride solution with a concentration of 0.5 mol/L, stirred by a constant temperature magnetic heating mixer for about 1 h, filtered by vacuum, and dried in the oven at 106 °C for 6 h. According to the procedure, the second pyrolysis was heated to 500 °C at a rate of 8 °C/min [47], and after reaching the experimental setting of 500 °C, the temperature was maintained constant and continued to be heated for 1 h. After natural cooling, the magnesium-modified biochar from rice straw and corn stover was prepared and named MRBC-500 and MCBC-500, respectively, and the prepared magnesium-modified biochar samples were stored in a self-sealing bag in a dryer to be used [48]. The naming of the biochar used in this experiment is shown in Table 2.

### 4.2. Determination of Biochar Properties

#### 4.2.1. Yield

After the biochar is impregnated with the magnesium chloride solution and dried, a certain mass is weighed with an electronic balance, and the mass is denoted as *M*_1_. The mass is put into the MFLGKDF406-12 tube furnace for re-pyrolysis. The mass after pyrolysis is weighed and denoted as *M*_2_. The yield of the modified biochar was

(1) $$H_{D}=\left(M_{2}÷M_{1}\right)\times 100\%$$

where $H_{D}$ indicates the calculated biochar yield; $M_{1}$ indicates the mass of biochar weighed before pyrolysis (g); $M_{2}$ indicates the mass of residual fraction after pyrolysis (g).

#### 4.2.2. Ash Content

Mg-modified biochar lg (accurate to 0.01 g) after passing through a 100-mesh sieve was weighed and placed in an MFLGKDF406-12 tube furnace at 800 °C for complete ashing for 4 h, and then removed and weighed after natural cooling. Calculate ash content by mass change before and after burning:

(2) $$D_{h}=\left(m_{1}-m_{2}\right)÷m_{3}\times 100\%$$

where $D_{h}$ indicates the calculated biochar ash result; $m_{1}$ indicates the ash and crucible mass (g); $m_{2}$ indicates the empty crucible mass (g); $m_{3}$ indicates the mass of biochar weighed for ashing (g).

#### 4.2.3. pH

The prepared Mg-modified biochar and distilled water were fully mixed in the ratio of 1:10 by mass and stirred well for 1~2 min, and the pH value of the Mg-modified biochar was determined by a pH meter after standing for half an hour [49].

### 4.3. Laboratory-Simulated Soil Culture Experiment

#### 4.3.1. Soil Sampling

The soil sampling location is situated within the premises of Nanning Arboretum, Guangxi. Guangxi Nanning Botanical Garden is dominated by a low-hill landscape, with a southern subtropical monsoon climate and conditions characterized by a relatively ample supply of water and heat. The soil here is red soil with a thick layer, and the altitude is about 210 m. The tree species is Tailed Giant Eucalyptus II, the forest is 3 years old, the row spacing is 2.0 m × 3.5 m, and the vegetation cover of the stand is about 75% [50]. Soil specimens were gathered on 4 December 2022. According to the S-shape method, soil samples from several random points at a depth of 0~20 cm were dug within a 20 m × 20 m square of Eucalyptus macrophylla plantation. After the sampling was completed, the fresh soil was screened using a 2 mm sifter and air-dried for an indoor simulated soil cultivation test [51]. After simple index determination, the basic properties of the sampled soil are shown in Table 3.

#### 4.3.2. Laboratory-Simulated Soil Experiment

Similar to the study of Kavitha, B, both magnesium-modified biochar and unmodified biochar were dried and set aside [52]. Weigh 150 g of air-dried soil into 500 mL white plastic culture bottle and thoroughly mix the prepared biochar (MRBC-500 and MCBC-500) with the soil at 0.5%, 1% and 2% (*w*/*w*) supplemental levels, respectively. Maintain the permeability of the culture environment by rehydrating every 2 weeks or so with distilled water to a soil weight that should contain 60% of the saturated water holding capacity. The sample was incubated in an incubator at a controlled temperature of (25 ± 1) °C, while a blank control group (CK) was established as the reference, without the addition of biochar. Three replicates were set up for each treatment, three groups in total. The soil numbers of the treatments followed the same nomenclature as that of biochar, and water was replenished by the weighing method every 2 weeks. The pH value, organic matter, available potassium, alkali-hydrolysable nitrogen, available phosphorus, total nitrogen and total phosphorus contents of the soil were measured 150 days after culture [53]. A total of 39 treated soil samples are listed in Table 4.

### 4.4. Methods for Determining Soil Properties

The analytical methods adopted in this test refer to some of Lu Rukun’s methods for the determination of basic properties of soil chemistry [54]. Specific soil testing methods and experimental equipment are shown in Table S1.

#### 4.4.1. Soil pH

The pH of the soil was determined by the potentiometric method; that is, 20 g of soil was weighed into a 50 mL beaker, 20 mL deionized water was added, and the suspension was determined by a pHS-25 pH meter after a 0.5 h shock.

#### 4.4.2. Soil Available Potassium

The ammonium acetate extraction-flame spectrophotometer method was adopted to determine the quick-acting potassium content of the soil. Weighing 5 g of soil sieved through a 12 mm sieve, 50 mL of ammonium acetate solution (1 mol/L) was prepared, and the two were fully mixed and shaken for 30 min, then left to stand, filtered and analyzed on the machine. The quick-acting potassium content was calculated by the following formula:

(3) $$K=v\times v_{0}/m$$

where K indicates the content of fast-acting potassium in the soil sample (mg/mL); v indicates the volume of liquid to be tested that is drawn up (mL); $v_{0}$ indicates the number of milliliters of impregnant added; and m represents the mass of air-dried soil (g).

#### 4.4.3. Soil Available Phosphorus

Sodium bicarbonate leaching and molybdenum-antimony resistance colorimetric methods were used to determine the absorbance of the filtered filtrate by leaching the soil with NaHCO_3_ [55]. It is calculated by the following formula:

(4) $$W_{p}=c\times V/\left(m\times ts\right)$$

where $W_{p}$ indicates the available phosphorus content; c indicates the concentration of phosphorus in the sample liquid calculated from the standard curve obtained by statistics (ug/mL); V indicates the colored liquid’s volume, 50 mL; m indicates the weight of the sample (g); ts indicates the partition multiple, 10 mL/10 mL = 1.

#### 4.4.4. Soil Nitrate Nitrogen

The potassium chloride leaching-flow analysis method was adopted. In this experiment, the absorbance was measured at 220 nm and 275 nm by preparing a standard solution of nitrite nitrogen for colorimetry. The soil nitrate-nitrogen content can be measured by the following formula:

(5) $$W\left(NO_{3}^{-}-N\right)=c\times V\times ts/m$$

where $W\left(NO_{3}^{-}-N\right)$ indicates the $NO_{3}^{-}-N$ content in the treated sample (mg/kg); c indicates the concentration of $NO_{3}^{-}-N$ in the sample solution calculated by the statistical standard curve (ug/mL); V indicates the volume of the liquid to be measured in the experimental scheme (mL); ts stands for partition multiple = 50/(2 ~ 10); m indicates the dry soil sample quality (g).

#### 4.4.5. Soil Ammonium Nitrogen

The potassium chloride leaching-flow analysis method was adopted. In this experiment, the absorbance was measured at 625 nm by the potassium chloride leaching and flow analysis method, and the content of soil ammonium nitrogen was calculated by the following formula.

(6) $$W\left(NH_{4}^{+}-N\right)=1000\times c\times V\times ts/m$$

where $W\left(NH_{4}^{+}-N\right)$ indicates the $NH_{4}^{+}-N$ content in the treated sample (mg/kg); c indicates the concentration of $NH_{4}^{+}-N$ in the sample solution calculated by the statistical standard curve (ug/mL); V indicates the volume of the liquid to be measured in the experimental scheme (mL); ts stands for partition multiple = 50/(2 ~ 10); m indicates the dry soil sample quality (g).

#### 4.4.6. Soil Total Nitrogen

The method of concentrated sulfuric acid—perchloric acid deboiling—Kjeldahl nitrogen determination was used.

#### 4.4.7. Soil Total Phosphorus

The plant samples were dried and ground, weighed and boiled with sulfuric acid and hydrogen peroxide. After the volume of the boiled liquid was fixed, the total nitrogen was measured by a micro nitrogen analyzer, and the total phosphorus was measured by the molybdenum antimony resistance colorimetric method.

#### 4.4.8. Soil Organic Matter

The hydrothermal potassium dichromate oxidation-colorimetric method was used. Mix 10 mL of potassium dichromate solution with 10 mL of concentrated sulfuric acid and shake continuously to mix well. After shaking for 30 min, let it stand for 20 min, add 10 mL water and finally, absorb 15 mL of the resting liquid into a 50 mL volumetric bottle to determine the absorbance at 590 nm wavelength.

### 4.5. Data Processing Method

Firstly, the experimental data obtained on the computer was sorted into Excel, and the simple processing of Excel statistics, summary tables and comparative analysis were used. For the sample data between different addition amounts and the sample data of different biochar species, the difference test was conducted by SPSS20.0. In addition, the tabular data obtained through the experimental formula calculation was rendered by Origin2021 plotting.

## 5. Conclusions

In this paper, rice and corn straw were completely or partially pyrolyzed under high temperatures and hypoxia to produce biochar. To enhance the value-added potential of rice straw and optimize the production performance of rice-straw biochar, this study prepared MgO-modified biochar (MRBC and MCBC), treated soil samples with different additive amounts and tested the soil’s pH value, available potassium, available phosphorus, alkali-hydrostatic nitrogen, total phosphorus, total nitrogen and organic matter contents after indoor simulation cultivation. The effects on soil chemical properties were analyzed. The findings indicated that following the utilization of biochar prepared from rice straw and corn straw, the chemical properties of soil samples were significantly improved compared to the control group, the soil pH value was increased, the contents of available potassium, available phosphorus and alkali-hydrolyzed nitrogen were increased, and the contents of total phosphorus, total nitrogen and organic matter were also increased. Among the biochar-treated soil indicators, only magnesium-modified biochar (MCBC and MRBC)-treated soils showed an increase in the total nitrogen content and organic matter content with increasing biochar additions. Under the same additional amount, the overall effect of Mg-modified biochar on improving soil fertility was better than that of unmodified biochar. Rice-straw biochar is better than corn-straw biochar in soil improvement; its yield is relatively high, and its preparation cost is relatively low. The soil improvement effect of Mg-modified biochar derived from rice straw surpasses that of Mg-modified biochar derived from corn straw in terms of available potassium, ammonium nitrogen, nitrate nitrogen, total phosphorus and total nitrogen content. In general, the application of biochar generally leads to improvements in soil chemical properties and enhancements in soil fertility, and different biomass types of biochar and different amounts of biochar have significantly different effects on soil properties, so different choices can be made, according to the requirements of forestry and agricultural production, laying a certain foundation for better utilization of biochar’s value.

## Figures and Tables

**Table 1:** Basic indicators of biochar.

Biochar Type	Yield %	pH	Ash Content
MCBC	61.65 ± 1.45	8.55 ± 0.05	11.07 ± 0.04
MRBC	70.25 ± 0.35	10.1 ± 0.15	36.47 ± 0.18

**Table 2:** Nomenclature of Mg-modified biochar.

Serial Number	The Raw Material	Pyrolysis Temperature (°C)	Continuous Pyrolysis Room (h)	Impregnation Concentration of MgCl_2_ (mol/L)
RBC-300	Rice straw	300	1	0.5
MRBC-500	Rice straw	500	1	0.5
CBC-300	Corn stover	300	1	0.5
MCBC-500	Corn stover	500	1	0.5

**Table 3:** Basic physical and chemical properties of the soil tested.

pH	Available Potassium Content (mg/kg)	Available Phosphorus Content (mg/kg)	Alkali-Hydrolyzed Nitrogen Content (mg/kg)	Total Nitrogen Content (g/kg)	Total Phosphorus Content (g/kg)	Organic Matter Content (g/kg)
3.43 ± 0.2	49.50 ± 1.5	3.35 ± 0.3	162.33 ± 3.0	1.22 ± 0.2	0.15 ± 0.0	35.89 ± 0.5

The water content of the soil is 15.63 ± 3.0%.

**Table 4:** Sample numbers of simulated indoor soil cultivation experiment.

Indicators	Processing Method (Type-Amount Added-Repeat)	Group	Indicators	Processing Method (Type-Amount Added-Repeat)	Group
1	CBC-0.5-1	A_1_	21	RBC-0.5-3	C_3_
2	CBC-0.5-2	A_2_	22	RBC-1-1	C_4_
3	CBC-0.5-3	A_3_	23	RBC-1-2	C_5_
4	CBC-1-1	A_4_	24	RBC-1-3	C_6_
5	CBC-1-2	A_5_	25	RBC-2-1	C_7_
6	CBC-1-3	A_6_	26	RBC-2-2	C_8_
7	CBC-2-1	A_7_	27	RBC-2-3	C_9_
8	CBC-2-2	A_8_	28	MRBC-0.5-1	D_1_
9	CBC-2-3	A_9_	29	MRBC-0.5-2	D_2_
10	MCBC-0.5-1	B_1_	30	MRBC-0.5-3	D_3_
11	MCBC-0.5-2	B_2_	31	MRBC-1-1	D_4_
12	MCBC-0.5-3	B_3_	32	MRBC-1-2	D_5_
13	MCBC-1-1	B_4_	33	MRBC-1-3	D_6_
14	MCBC-1-2	B_5_	34	MRBC-2-1	D_7_
15	MCBC-1-3	B_6_	35	MRBC-2-2	D_8_
16	MCBC-2-1	B_7_	36	MRBC-2-3	D_9_
17	MCBC-2-2	B_8_	37	CK-1	E_1_
18	MCBC-2-3	B_9_	38	CK-2	E_2_
19	RBC-0.5-1	C_1_	39	CK-3	E_3_
20	RBC-0.5-2	C_2_			

## Data Availability

Data are contained within the article and Supplementary Materials.

## Supplementary Materials

The following supporting information can be downloaded at: https://www.mdpi.com/article/10.3390/molecules29143268/s1, Table S1. Test methods for soil properties. Table S2. Comparison of findings.
