# Supplementary material for: Study on the Effect of Magnesium Chloride-Modified Straw Waste Biochar on Acidic Soil Properties
Source: Molecules. 2024 Jul 10;29(14):3268. doi: 10.3390/molecules29143268 (PMC11278922; doi:10.3390/molecules29143268)
Supplement: Supplementary file 1 [file molecules-29-03268-s001.zip › molecules-3077027-supplementary.pdf]

Supplementary Material

Study on the Effect of Magnesium Chloride-Modified Straw Waste Biochar on Acidic Soil Properties

Zhigao Liu [1,2,3], Yuhang Dai [1,3], Tianyi Wen [2,*], Penglian Wei [2,*], Yunlin Fu [2] and Mengji Qiao [2]

[1] College of Resources, Environment and Materials, Guangxi University, Nanning 530004, China; lzgk18@gxu.edu.cn (Z.L.); 18014389048@163.com (Y.D.)
[2] College of Forestry, Guangxi University, Nanning 530004, China; fylin@126.com (Y.F.); qiaomengji1982@163.com (M.Q.)
[3] State Key Laboratory of Featured Metal Materials and Life-Cycle Safety for Composite Structures, Guangxi University, Nanning 530004, China
* Correspondence: gxustu1987@126.com (T.W.); weipenglian@gxu.edu.cn (P.W.)

Table S1 Test methods for soil properties

Indicators	Methods	Equipment	Types
pH value	Mixed indicator colorimetry/ Potential method	Ph meter	Phs-25
Organic matter	Volumetric method using potassium dichromate	Enzyme-labeled instrument	INFINITE M200 Pro
Total nitrogen	Potassium dichromate-sulphuric acid digestion	Continuous flow analyzer	PROXIMA
Nitrogen hydrolysis	Alkaline diffusion		
Total phosphorus	Sulphuric acid-perchloric acid decoction	UV spectrophotometer	UV-2500
Quick-acting phosphorus	Sodium bicarbonate method	UV spectrophotometer	UV-2500

Quick-acting potassium	Ammonium acetate-flame photometric method	UV spectrophotometer	UV-2500
Total potassium	Sodium hydroxide melt-flame photometric method	UV spectrophotometer	UV-2500
Cation exchange capacity	EDTA-ammonium rapid method	UV spectrophotometer	UV-2500
Chloride ion	Nitrate titration		
Effective Boron	Curcumin colourimetric method		

Table S2 Comparison of findings

**Indicators**	**This experiment MCBC**	**This experiment MRBC**	**MCBC-500**	**MRBC-500**
pH value	6.58	6.87	3.93	3.62
Quick-acting potassium （mg/kg）	179.45	240.14	172.5	195.3
Quick-acting phosphorus(mg/kg)	10.83	14.66	7.69	9.53
Nitrate (mg/kg)	157.74	165.34		
Ammonium nitrogen (mg/kg)	58.47	62.51		
Total phosphorus (mg/kg)	173	186	225.34	212.69
Total nitrogen (g/kg)	1.3	2.14	1.18	1.35
Organic matter (g/kg)	29.43	34.57	25.82	22.43

References:

Liu, Z.; Yuan, D.; Qin, X.; He, P.; Fu, y. Effect of Mg-Modified Waste Straw Biochar on the Chemical and Biological Proper-ties of Acidic Soils. Molecules. 2023, 28, 5225.
